# On Puerperal Insanity

**Published:** 1848-10-01

**Authors:** 


ART. IV.—*On Puerperal Insanity.* By JAMES MACDONALD, M.D., formerly Physician to the Bloomingdale Asylum for the Insane, and one of the Attending Physicians of the New York Hospital. New York, 1848.

HAVING so recently published in Nos. I. and II. of this journal, an able and elaborate essay on puerperal insanity, from the pen of Dr. J. Reid, some apology is due to our readers for again entering upon the consideration of this subject. Having received through the kindness of Dr. Macdonald, of New York, a copy of his essay on puerperal insanity, we feel it our duty to submit to the profession at once, this distinguished physician's views of this important form of mental derangement. The author holds a high position in America, and has had great experience in the treatment of mental affections, and his opinion is much valued by all thinking men on the other side of the Atlantic. We may therefore consider this essay as conveying to the English reader a fair *résumé* of the views of the American faculty of the pathology and treatment of puerperal insanity. Dr. Macdonald treats the malady as it occurs during the three periods of pregnancy, parturition, with its consecutive state, and lactation, considering that these three physiological stages are intimately connected together, forming parts of one whole, and being but links of one chain.

In reference to insanity occurring during lactation, Dr. Macdonald remarks that in a given number of cases it originated in 17 out of 66 cases. He thinks the disease of slow progress and gradual development. He says, "When it shall be better known, and its incipient stage early recognised, it will doubtless be often practicable to prevent it altogether, or to stop its further progress when first observed. It generally occurs in women of delicate organization who have borne children rapidly and suckled them for a long time, and is usually preceded by loss of flesh, strength, and spirits—accompanied by marked pallor of the countenance. The uniform occurrence and persistence of these symptoms prior to the development of insanity, is remarkable. When the secretion of milk and the strength of the mother begin to fail, it is usual to resort to stimulants in the form of porter, wine, milk-punch, &c.,—but these, affording only temporary relief, the malady advances with increased rapidity. Emaciation, paleness, languor and lowness of spirits, are more and more apparent—restlessness, sleeplessness, changeableness and irritability of temper, supervene; the patient feels unequal to her usual efforts, and unlike herself; she begins to think, and her reflections almost invariably assume a sombre hue, and soon concentrate on a single subject, and that subject is almost invariably herself. She thinks of herself only in connexion with impending death, or with reproach for having been an undutiful wife, a careless mother; or for having failed to perform the chief relative duties of life. Her attention now seizes on a single subject and fixes on it with that tenacity so remarkable in melancholy and monomania. This consideration of herself often leads to the belief that she has committed the unpardonable sin—that all her future hopes are cut off, and she not unfrequently attempts suicide. It is probable that women who commit infanticide of their own offspring, are often in this state of mind. I would not be understood, however, to say that all women who become deranged while suckling, are melancholy, but that the great majority are so."

Dr. Ashwell refers these functional results occurring during suckling, to an impaired and attenuated condition of the blood, and a consequently depressed state of the nervous system, especially of the organic system of nerves.*

Does insanity occur most frequently during *pregnancy*, the *puerperal state*, or at the *period of lactation?* Our author observes, that of the 66 cases which have come under his notice, 40 occurred at the period of pregnancy; 44 took place at the strictly puerperal period (including one where the disease developed itself during labour); 18 at the time of lactation. He limits the puerperal cases to the first month.

Dr. Macdonald considers the malady in its *acute* and *chronic* stages.

"1. *The acute* is of recent origin, characterized by great febrile excitement, frequent pulse, hot skin, incoherence, raving, unceasing jactitation, and sleeplessness. It is dangerous to life; it is mistaken for phrenitis, and is often fatal under any management, but particularly when treated actively. This acute form of the disease almost uniformly occurs soon after delivery, during the strictly puerperal state, though it sometimes happens during lactation.

"2. *The Chronic stage* is where the disease is of several weeks standing, or where it has come on gradually. It is without febrile excite-

* Guy's Hospital Reports.

ment — the pulse is slow—the skin cool — the mind may be equally incoherent, as in the acute stage; or the patient may be labouring under monomania or dementia."

He considers the disease "probably of much more frequent occurrence than is generally supposed. Esquirol says, the number of women who become insane after parturition, and during or after lactation, is much more considerable than is usually believed. In fact, nearly one-twelfth of the insane women received into the Salpêtriere, have become so under these circumstances. Some years, this proportion amounted to one-tenth; thus, of 1119 females admitted into the insane division, in the years 1811, 1812, 1813, 1814, ninety-two became deranged after parturition, or during or immediately after lactation; and of these ninety-two women, sixty belonged to the years 1812 and 1813, during which time there were but six hundred admissions. And if from this total number of insane females admitted in the course of these four years, we deduct all who had passed the age of fifty, beyond which epoch they are not exposed to the influences of parturition and lactation, we shall arrive at the conclusion, that insanity, following child-birth and lactation, is much more common than was formerly supposed. This is true, particularly among the rich. The proportion in this class is, according to Esquirol's observation in his private practice, about one-seventh. But it is also true, that cases of mental derangement after weaning are rare among the rich, while they are common among the poor, whether these wean their children voluntarily or by compulsion. The precautions which those who are in easy circumstances have it in their power to take, explain this difference.

"Of six hundred and ninety-one females admitted into the Bloomingdale Asylum, forty-nine became insane during pregnancy, parturition, and lactation; making one in every fourteen, or seven and one-tenth per cent. of females of all ages. If we take into account the great number of women beyond the age of child-bearing, and the unascertained causes of insanity, the importance of pregnancy, parturition, and lactation, in causing insanity, will be quite evident. Like mental derangement arising from other causes, puerperal insanity assumes different forms. Of the sixty-six patients whose cases are here recorded, including those admitted into the Bloomingdale Asylum, thirty-four were labouring under mania, twenty-eight under monomania, and four under dementia. Of the ninety-two cases reported by Esquirol, forty-nine were instances of mania, thirty-five of monomania, and eight of dementia."

The causes Dr. Macdonald considers as *predisposing* and *exciting*. The first question that presents itself relates to the former. How often is there a family predisposition, either hereditary or acquired? In 17 of the 66 cases, there was an hereditary or family predisposition, and in 7 an acquired predisposition from former attacks of insanity. *One* was said to have been hysterical, and *one* to have great nervous susceptibility. This makes almost 26 per cent. of cases in which there is an hereditary or constitutional predisposition. Besides hereditary, the other predisposing causes are extreme susceptibility, and former attacks of insanity.

Dr. Gooch observes, that a large proportion of the cases of puerperal insanity occurred in patients in whose families disordered minds had already appeared. The patients, too, were of a susceptible disposition, nervous, remarkable for an unusual degree of that peculiarity of nerve and mind which distinguishes the female from the male constitution. It may be laid down as an axiom, that in rather more than 1 of every 4 cases of puerperal insanity, there exists a family predisposition. In some, this predisposing cause is so strong, that it needs only the recurrence of natural labour or of lactation to bring on another attack of mental disorder.

It may be expected, that should any moral causes occur during the puerperal state, they would, owing to the then more impressible state of the nervous system, have a much more serious influence on the mind than at any other period. As far as Dr. Macdonald could ascertain, 12 out of the 66 cases which have been under his care, were influenced by moral causes, and these were not all so decided as to be considered *the cause.* They were mostly viewed as incidental or co-operative. There exists in one of the towns of Holland, a municipal regulation, which orders that a mark shall be put on every house in which there is a recently confined female. This mark serves as a safeguard against the visits of constables and police agents, which on the continent of Europe are the sources of great terror. In ancient Rome, it was customary to suspend a crown over the door of houses of females in this state, in order to let it be known that such residence was to be kept sacred from intrusion.

Of 92 cases of puerperal insanity reported by Esquirol, 46 (exactly one half) became deranged after being exposed to strong moral influences.

On the subject of physical causes, Dr. Macdonald observes:—

"Of the 66 cases observed by myself, 6 only are reported as having been influenced by physical causes. By this I mean, of course, physical causes having no connexion with the puerperal state, but entirely extraneous. Of these, the most frequent was cold,—the exposure of the patient to cold air or cold water. But this exposure to cold, besides being an occasional *cause*, is often one of the *effects* of insanity, from an instinctive desire of maniacs to expose their bodies to the open air and cool water. It is frequently one of the first acts of insanity. The secretion of milk and the lochial discharge being causes strictly connected with the puerperal state, will be adverted to when we come to the pathology of the disease.

"The disordered state of the stomach, bowels and liver, which sometimes ensues during pregnancy, is not uncommonly an exciting cause.

"Besides the causes which have been enumerated, there are certain circumstances which are supposed to exert a marked influence in developing the disease; such as the birth of the first child; the age of the patient, and the period after confinement.

"Of our 66 patients, 29 became insane with their first children; 9 with their second; 6 with their third; 9 with their fourth; 2 with their fifth; 2 with their sixth; 1 with her seventh; 3 with their ninth; 1 with her tenth, and 4 unknown. It is also quite probable that some of these now put down as having become insane with their second, third, fourth, &c., children, may have been so with their first ones. It is therefore evident that by far the largest proportion of cases occurs with first children.

"*Age.*—Of the 66 cases, 2 were under 20 years of age; 45 were between 20 and 30; 11 between 30 and 40; 7 between 40 and 44, and 1 unknown."—pp. 137, 138.

It appears, of the author's 66 cases—

"Twenty-nine became deranged within one week after parturition, and 15 during the subsequent three weeks; making altogether, 44 cases during the first month, or strictly puerperal period. During the second month, 5 became insane; during the third and fourth months, 4; at 6 and 10 months each, 2; at 9 and 15 months each, 1 became deranged; 3 became insane during lactation, without there having been any precise time specified; 1 while labour was going forward, and 4 in the course of pregnancy. Thus it is seen, that during the first month, or strictly puerperal period, 44 out of 66 of the cases, (a very large proportion,) originated; that in the course of the second month, which may still be regarded a puerperal period, 5 more originated, and that the proportion increases from day to day, as we approach the day of parturition, and diminishes as we depart from it."—p. 139.

In considering the causes of this form of derangement of mind, which may be termed strictly *puerperal*, Dr. Macdonald remarks:—

"In viewing these matters, two important and interesting questions arise: how is the secretion of milk affected? and how is it with the lochial discharge? Different observers have assigned to these things, particularly to the milk, very unequal degrees of importance. Some have looked to this as the sole cause and essence of the disease. The older physicians believed that milk was translated to the brain, and they attributed to metastasis all the symptoms following its diminution or suppression. Leveret asserted that veritable milk could be found within the cranium; Boerhaave thought that all the different symptoms which manifest themselves after child-birth as distinct diseases, depend on one and the same cause, though manifested in various ways, and should be treated in the same manner. Most of the older obstetricians attributed the various maladies supervening on child-birth and nursing, to the metastasis of milk. This is still the popular belief. But recent observation has demonstrated that milk is not found effused in the abdominal cavities of puerperal women who have died of peritonitis. Neither is it found within the crania of those who have died insane. Modern pathologists think that after confinement and during lactation, there exists what is called a milk diathesis, which modifies and characterizes all the secretions; that the excessive susceptibility of puerperal women, and nurses, renders them more subject to external influences; that these influences, acting on different organs, cause the development of diseases peculiar to those organs—diseases which are modified by the prevailing milk diathesis.

"Esquirol says sometimes the milk is totally suppressed—at others, only diminished; while at other times, insanity is developed without either suppression or diminution of milk, and while the infant is constantly improving under its use. He asks also the question, is the suppression or diminution of milk, the cause or effect of mental derangement? and answers, that insanity is most frequently developed in females who *do not* suckle their children.* He adds, that the majority of facts go to show that the milk is either suppressed, diminished, or changed in quality, previous to the development of insanity; but that on the other hand, there are cases in which mental derangement is manifested previous to any change in the secretion of milk. In looking over the cases," says Dr. Macdonald, "recorded both by myself and others, I find a deficiency on this point. Authors give no numbers whatever. And of the 66 here reported, there are only 40 in which the secretion of milk and the lochial discharge are mentioned, either separately or together,

* Of his 92 cases, 63 were married and 29 unmarried women. The single women, he observes, rarely nurse their children.

either directly or indirectly. Of these 40, it is stated that neither the lochia nor milk were suppressed in 19; making nearly half of the whole number in which this secretion and this discharge were alluded to, when these were not affected prior to the development of insanity. In thirty cases, the secretion of milk was not checked prior to the development of insanity, and in 19 cases the lochial discharge was natural. In 4 cases the milk was checked after the occurrence of mental disorder; in 1 case the milk was scanty. So there remain of the 40, only 6 cases in which the milk, and 3 in which the lochia were checked or suppressed, before the occurrence of insanity. These, imperfect as they may be, are the only numbers or real facts relating to the subject, that I can find; and their bearing on the pathology of the disease is, that the lochia and the suppression of milk have less to do in originating it than is generally supposed. But though the suppression of milk may not so often cause insanity as some have believed, yet that change which is wrought in the whole system by the secretion of milk, the milk diathesis as it has been termed, may in all cases be the essential cause of the disease.

"Esquirol says that sudden weaning, whether voluntary or compulsory, is the cause of insanity, when nurses neglect those precautions which prudence and experience dictate. In 19 of his 92 cases, insanity was manifested a few days or immediately after weaning, and following imprudence or negligence. Insanity after weaning is rare among women in easy circumstances, because they have the means of taking good care of themselves. As to the influence of weaning, in causing puerperal insanity, I know of no facts corresponding with those of Esquirol. Of my own 66 cases, not one arose from this cause; but weaning is not an uncommon sequel of the disease. Gooch says, 'among the fashionable women of London, nothing is so common as not to nurse their children; the milk comes in about one or two days after delivery, and the breasts become as hard as stones, but not a drop is extracted; and sometimes by cold spirit lotions applied to the breasts, &c., the milk is suppressed in a few days.' He knew 'more than a hundred instances treated in this way during the first week after delivery—a time much more liable to a disordered mind than a later period, and in not one did it occasion puerperal insanity.' These observations not only show that weaning is not a cause of insanity in women in easy circumstances, but that the suppression of the milk during the strictly puerperal state, within a few days after delivery, has not so much influence in the production of mental disorder as has been conjectured."

Dr. M. considers that puerperal insanity may originate independently of all these causes—that it is a disease strictly of the puerperal period.

In reference to the pathology of the disease, our author observes, that when insanity occurs during pregnancy, there generally exists, as a leading physical symptom, fulness and congestion of the vessels of the head. The disease, however, Dr. Macdonald considers to be more the result of *irritation* than inflammation of the brain. Phrenitis occurring during the puerperal period is sometimes mistaken for true puerperal insanity; and unless active measures are had recourse to, the patient will slip through our fingers.

"If we justly estimate the symptoms of this disease, as we should do those of any other form of mental alienation, without the ever-present idea of inflammation, we shall find the physical phenomena corresponding with the previous state of the system. In the acute form or stage of puerperal insanity, we shall find the pulse frequent; perhaps from 120 to 140—but feeble like the pulse of a typhoid patient; in some cases, however, it may be full and strong, but these are exceptions which prove the rule. We shall find the head and surface generally hot, but it is the heat of febrile excitement, and is sometimes accompanied by cold extremities—we shall find great jactitation and restlessness, with perhaps subsultus—the tongue coated and foul, and sometimes dry, and red or brown, the bowels constipated, and all the secretions depraved. These are the leading symptoms of the acute stage. In the chronic stage there is an entire absence of all febrile as well as inflammatory symptoms."—pp. 144, 145.

Dr. Macdonald asks—

"Is there anything in the character of puerperal insanity to distinguish it from other forms of madness? In the acute form of the mania, which succeeds parturition, we observe an intensity of mental excitement, an excessive incoherence, a degree of fever, and above all, a disposition to mingle obscene words with the broken sentences; things which are rarely noted under other circumstances. It is true that in mania, modest women use words, which in health are never permitted to issue from their lips—but in puerperal insanity, this is so common an occurrence, and is done in so gross a manner, that it early struck me as being characteristic. And is there not a reason for it? Do not the disturbed uterine functions give rise to such ideas? In recent and acute cases of puerperal mania, there is greater febrile excitement, and greater frequency of pulse than is usually found in other kinds of acute mania. In fine, these more nearly resemble phrenitis than ordinary cases of mania. If two cases of mania were presented, the one arising during the puerperal state, and the other not, would it be possible, without any knowledge of their histories, to distinguish between them? We are not prepared to say that it would be in all cases, but think it might be in the more striking instances of this disease. Esquirol says the physiognomy of puerperal insanity has something so peculiar, that it can be recognised by those who are accustomed to treat it.

"Where insanity occurs during protracted lactation, it very generally assumes the form of melancholy—of lypemania or religious melancholy—of homicidal or suicidal mania; but it does not, under these circumstances, appear to take so destructive a character as puerperal mania?"—pp. 147, 148.

On the subject of *prognosis*, we find nothing new in Dr. Macdonald's essay. He quotes the aphorism of Dr. W. Hunter, who says, (speaking of women insane during the month,) "when out of their senses, attended with fever like paraphrenitis, they will in all probability die." Dr. Macdonald says—

"If we meet with a patient having a pulse of 120 and upwards, accompanied by great heat, restlessness, sleeplessness, constant jactitation, perfect delirium and incoherence of language, without a rational interval, we may predict a fatal result. If typhoid symptoms be superadded to these, we may conclude a fatal result as much more certain."—p. 150.

Dr. Macdonald found among his own cases the per centage of recoveries to be $80\frac{1}{3}$—of the 53 cases who recovered under Dr. Macdonald's care, not one was restored during the first month. It would appear by a tabular statement contained in the essay, that—

"The same proportion recover within the first three months after attack, that do in the subsequent three months—that is to say, 17 during each period; making altogether 34 out of 53, in the course of the first six months—that in the next ensuing three months—that is, from the sixth and ninth months—the next highest proportion recover. After the expiration of a year, one recovered; after the expiration of two years, another; and after the long period of three years, one other.

"This corresponds very nearly with Esquirol's results—two-thirds of whose recoveries took place within the first six months after attack. Hence, in favourable cases we may predict recovery in three months. But if the patient pass by that period, we may expect it during the subsequent three months."—pp. 151, 152.

On the subject of treatment, Dr. Macdonald lays down the general rule, that the

" True method of proceeding is to carefully observe each case separately, to examine well the symptoms, and finding out the functions and organs most disordered, prescribe accordingly."—p. 152.

He divides the disease into two stages—the *recent* and *chronic*. He says—

" These stages do not always depend upon the length of time the patient has been ill; they rather mark the character of the disease, and have been already described. The first is attended with great danger under any treatment, and if life be saved, it is sometimes at the expense of reason. The *second*, if properly treated, is attended perhaps with little danger, either as respects life or continued insanity."—p. 152.

General bleeding, our author says, is seldom necessary, particularly in public institutions. Referring to the necessity for venesection, Dr. Macdonald says—

" The symptoms, in the early stages of puerperal mania, are sometimes so deceptive and so simulate those of phrenitis, that practitioners are led to draw blood in large quantities. That venesection is occasionally useful, there can be no doubt. I can imagine it so even in some cases of delirium tremens, because the most judicious practitioners have found it of advantage—but in the great majority of instances of puerperal mania, it will only tend to increase the delirium and endanger life. Muttering or violent delirium, heat and tossing about of the head, contraction of the pupils, a frequent pulse, constant jactitation of the body, with movements of the limbs, and a dry tongue, constitute a group of symptoms indicative of what is called in the books, inflammation of the brain—but of a species of inflammation, if inflammation it be, which may be better treated by anodynes and stimulants than by blood-letting. Abercrombie mentions a variety of inflammation of the brain, in which venesection is fatal and wine useful. Some forms of puerperal mania resemble it. Finally, we should bleed, not because there is frequent pulse and violent delirium, but because there exists some good reason for so doing; as, for example, when the patient is naturally vigorous and plethoric—has been suddenly seized, has a full, hard pulse, great heat, and has suffered cerebral congestion, prior to the development of insanity.

" Local bleeding by cupping and leeching, is more frequently admissible than general blood-letting. Seven of the sixty-six patients that have been under my care, were cupped, aud four leeched. The cupping is chiefly employed to relieve congestion of the brain—the glasses being applied to the temples and the occiput. Cupping is also performed over the sacrum, and leeches applied to the vulva and thighs to irritate and invite the flow of the menses.

" When blood-letting is required, cupping and leeching answer the purpose in almost every case; but even these have been seldom called for in the cases which I have had to treat."—pp. 153, 154.

When the force of the circulation has been reduced, the next indication is to evacuate the stomach and bowels, and restore the secretions to a healthy state. For this purpose, our author recommends calomel in full purgative doses, or in doses of from five to eight grains, at bed-time, followed, next morning, by castor oil, or the compound infusion of senna, or the rhubarb and magnesia mixture. The calomel should be combined either with the extract of hyoscyamus or conium. The bowels should be entirely relieved of their accumulation, and the biliary and other secretions restored to their natural and healthy state.

"When the patient is feeble, small doses of hydrarg. c. creta and blue pill, may be used in place of calomel. Neither of the mercurial preparations, however, should be carried so far as to affect the system or cause ptyalism, for this might produce so much constitutional irritation as to affect most unfavourably the nervous system. But I regard calomel and blue pill, particularly the latter, when cautiously exhibited as an alterative cathartic, as among the most valuable remedies in the treatment of this disease. I would not, however, be understood to advise these remedies as a matter of course, because the patient has puerperal insanity, or as a specific; but to remove a disordered state of the stomach, liver and bowels, a condition which, so long as it exists, must have a most unfavourable influence on the nervous system. Mercurials in this way have been given to at least half of the patients for whom I have prescribed. But in a single case was mercury exhibited for the express purpose of causing ptyalism. This was a case in which the prognosis was most unfavourable from the beginning, and in which the patient died before the system was affected. It was given as a dernier resort.

"After calomel and blue pill, the most useful cathartics, perhaps, are compound infusion of senna, castor-oil, rhubarb, and magnesia. Blue mass combined with colocynth, or colocynth and scammony to quicken its action, will be found a very useful cathartic. But I would by no means advise active and prolonged purging particularly when the patient has been much exhausted; and when there exists irritation of the mucous membrane of the stomach and bowels, cathartics should be avoided, and enemata used instead."—pp. 155, 156.

Having relieved the *head*, and attended to the condition of the *secretions*, the next important indication is to soothe the nervous irritation and promote sleep:

"Cold applications to the head, stimulating pediluvia and warm baths, will have this effect, and may be used at the onset of the disease. So may what are strictly speaking, anodyne and anti-spasmodic remedies; but these must be exhibited with caution. The anodynes and anti-spasmodics most in use are opium in some of its forms, conium, hyoscyamus, camphor, bella-donna, lupuline, assafœtida, and valerian. In selecting the particular one for use, regard must be had, not only to the patients' symptoms, but to their idiosyncrasies. We should inquire how opium affects them, and if it usually affects them unfavourably, it should not be exhibited; but if we are informed that opium usually has a soothing, soporific effect, then it should be given freely if the patient has been long without sleep, and is in a state of morbid susceptibility. It is generally advisable to give it at first in a large dose, (two or three times the size of the usual dose.) In my experience, *black drop* has been preferable to any other preparation. This has often appeared to me to quiet restlessness and to procure sleep when other preparations have failed. But the salts of morphia, opium in substance, laudanum and Dover's powders, may all be employed. Great heat and redness of the face and scalp, a full and hard pulse and contraction of the pupils, with symptoms of determination to the brain, contra-indicate the use of opiates. As a single symptom, perhaps no one points out more clearly the nature of the soporific to be employed, than the state of the pupil. If this be dilated, opium may be exhibited, if the other symptoms do not prohibit its use. It it be contracted, then one of those narcotics, the peculiar effect of which is to dilate the pupil, should be administered. As a general observation, perhaps no anodyne or narcotic has a more kindly influence in cases of puerperal mania, than a combination of camphor and hyoscyamus, given in doses of from *one* to *five* grains each of the gum and extract in the form of a pill—or the extract or tincture of hyoscyamus may be rubbed up with camphor julep, and the latter administered in doses of half to a whole drachm, three or four times in the course of twenty-four hours. Conium, lupuline, &c., may be given in the same manner with hyoscyamus. Assafœtida and valerian are particularly useful when hysterical symptoms prevail. Whichever preparation of opium or whatever other sedative may be selected, should be given in sufficiently large doses (triple, quadruple, or more if necessary,) to subdue nervous excitement; and when the proper quantity to produce this effect has been ascertained, it should be repeated every 6, 8, 12 or 24 hours, as may be found requisite to control this leading symptom. And it is the experience of the writer, that when this result has been obtained, it is not necessary to increase the dose, but that the disease generally yields, so that the quantity may soon be diminished, or the remedy altogether withdrawn."—pp. 156—158.

When there is much febrile excitement and determination to the brain, warm bathing is contra-indicated. It will be found most beneficial in the melancholic form of the malady, when the extremities are cold. In cases where the exhaustion is great, wine must be given. It will be found to lessen the frequency of the pulse, to act on the skin, and promote sleep. The cases in which wine is admissible

"Are characterized by a delirium so strongly marked, that the patient utters nothing connected or rational; all her thoughts appear incoherent—there is almost constant motion of the hands and feet, but these movements are as unceasing and apparently useless, as the words uttered are disconnected and unmeaning. There exists a peculiar sunken and haggard expression of countenance—the eye becomes dull, and there is every indication of a most prostrate condition of the nervous system. The pulse is frequent, (from 110 to 150,) while the impulse of the heart is very feeble. As in typhus THIS is in truth one of the most favourable conditions for the exhibition of wine; but on the contrary, when the pulse is weak, while the impulse of the heart is at the same time strong, stimulants will be found useless, if not decidedly injurious. Besides the above symptoms indicating the use of wine, general typhoid symptoms sometimes supervene—which of course render the necessity for administering stimulants greater."—p. 159.

Dr. Macdonald has no opinion of blisters as a means of counter-irritation in the recent stage, but when the symptoms are chronic in their character, he considers them most useful, applied either to the back of the neck, to any portion of the upper spinal region, and to the extremities. He speaks favourably of the application of tartar-emetic ointment and croton oil. He says in the treatment of chronic cases of puerperal insanity—

"The most valuable classes of medicines in this stage, are tonics combined with sedatives or anodynes, bark, sulph. quinine, sulphate and carbonate of iron—particularly the two latter. The iodide and citrate of iron may also be employed. These medicines are most serviceable when the patient is pale and ex-sanguine, the circulation languid, the digestion weak, and the mind feeble or melancholy. Large doses are not advisable, but small ones continued for a length of time are most useful. Thus I have found from five to ten grains of the carbonate, or $\frac{3}{4}$ to $1\frac{1}{2}$ grains of the sulphate sufficiently large; or one grain of the sulphate of quinine, combined with extract of hyoscyamus, or some other sedative, may be given three or four times a day. Shower baths may also be employed in this stage. These should in general though it often happens that the mind of the patient is entirely restored, while the menses remain quite suppressed. Whatever contributes to improve the general health, goes to re-establish the menses; but after the former has been restored, it often becomes necessary to use means for the re-establishment of the catamenia. Aloes in some form or combination is the most useful of these, and may be given in the form of pills with myrrh, &c., or in tincture with hiera picra. Hip baths and hot pediluvia may also be employed at the expected periods.

"Horse-back exercise and tonics may also be used with good effect. If the mind, however, be restored, we need not be so anxious for the return of the menses; for this result will generally, sooner or later, follow without the use of remedies."—pp. 160, 161.

In concluding our brief notice of Dr. Macdonald's essay, we may pronounce it to be an excellent *résumé* of what is generally known in reference to the form of mental derangement occurring during the period of utero-gestation. Although the author does not startle us by any new views as to the pathology and treatment of puerperal insanity, his dissertation is not the less valuable, containing, as it does, the result of the combined observation and experience of all who have paid particular attention to this class of affections.

